# Disclosure bias for group versus individual reporting of violence amongst conflict-affected adolescent girls in DRC and Ethiopia

**DOI:** 10.1371/journal.pone.0174741

**Published:** 2017-04-04

**Authors:** Lindsay Stark, Marni Sommer, Kathryn Davis, Khudejha Asghar, Asham Assazenew Baysa, Gizman Abdela, Sophie Tanner, Kathryn Falb

**Affiliations:** 1 Department of Population and Family Health, Columbia University Mailman School of Public Health, New York, New York, United States of America; 2 International Rescue Committee, New York, New York, United States of America; Stellenbosch University, SOUTH AFRICA

## Abstract

Methodologies to measure gender-based violence (GBV) have received inadequate attention, especially in humanitarian contexts where vulnerabilities to violence are exacerbated. This paper compares the results from individual audio computer-assisted self-administered (ACASI) survey interviews with results from participatory social mapping activities, employed with the same sample in two different post-conflict contexts. Eighty-seven internally displaced adolescent girls from the Democratic Republic of the Congo and 78 Sudanese girls living in Ethiopian refugee camps were interviewed using the two methodologies. Results revealed that the group-based qualitative method elicited narratives of violence focusing on events perpetrated by strangers or members of the community more distantly connected to girls. In contrast, ACASI interviews revealed violence predominantly perpetrated by family members and intimate partners. These findings suggest that group-based methods of information gathering frequently used in the field may be more susceptible to socially accepted narratives. Specifically, our findings suggest group-based methods may produce results showing that sexual violence perpetrated by strangers (e.g., from armed groups in the conflict) is more prevalent than violence perpetrated by family and intimate partners. To the extent this finding is true, it may lead to a skewed perception that adolescent GBV involving strangers is a more pressing issue than intimate partner and family-based sexual violence, when in fact, both are of great concern.

## Introduction

Violence against women and girls is one of the most prevalent human rights violations in the world [1]. Evidence shows that female survivors of physical or sexual violence are at increased risk for a range of poor sexual, reproductive, and mental health outcomes [2–9]. Violent experiences during adolescence may confer additional negative impacts throughout the life course, including lower educational attainment, less community engagement, and greater likelihood of living in poverty [10, 11].

Due to high levels of stigma and victim-blaming, attempts to measure GBV are highly sensitive to the data collection approach and its ability to mitigate nondisclosure. While global strides have been made in gender-based violence (GBV) prevention and response, the methodologies used to conduct evaluations, needs assessments and broader research on GBV, have attracted less attention and development.

These measurement challenges are particularly acute in conflict-affected settings, where women’s and girls’ vulnerabilities are often magnified [12]. Non-governmental organizations working in such contexts typically utilize existing data such as service provider records when conducting needs assessments [13–16]. Yet, these records are not representative of the wider population, omitting the experiences of survivors who do not actively seek or have access to help. Primary data, when collected, often rely on group-based qualitative discussions to assess protection concerns of girls and women [17–19]. To date, information about the validity of different methods to capture adolescent girls’ experiences of violence is limited.

Admittedly, there are ethical challenges in efforts to validate any means of measuring such a sensitive topic; measurement itself may have repercussions for participating girls or women, especially in fragile contexts. As such, the humanitarian community has not had the opportunity to systematically investigate potential measurement bias related to the use of commonly utilized qualitative group-centered methodologies with adolescents. If indeed such methodologies skew the data in some way, humanitarian actors’ efforts to design effective, targeted interventions for violence prevention and response may be impeded.

This paper provides insight into assessing GBV among adolescent girls by examining results from two methodological approaches that were utilized with the same sample in two post-conflict contexts. Both approaches were part of a larger baseline study to evaluate a violence prevention initiative targeting adolescent girls, and both approaches sought to answer the same research question: *What are the primary experiences of GBV among conflict-affected adolescent girls in Democratic Republic of Congo (DRC) and Ethiopia*? We were able to analyze and compare the results from quantitative individual audio computer-assisted self-administered (ACASI) survey interviews with results from qualitative social mapping activities among the same sample of conflict-affected adolescent girls to reveal discrepancies in the conceptualization and reporting of violence, and to ultimately recommend approaches likely to yield more valid data on interpersonal violence in humanitarian settings.

## Methods

### Participants

Study participants were drawn from a larger sample of 1788 girls and adolescents participating in the baseline evaluation across 14 conflict-affected communities in South Kivu, the DRC, and 3 (primarily Sudanese) refugee camps in Benishangul-Gumuz, Ethiopia. Overall eligibility for the larger evaluation was limited to 10-14-year-old female adolescents in the DRC, and 13-19-year-old female adolescents in Ethiopia. Languages of participants included Swahili, and Mashi in the DRC, and Funj, Regarig, Ingessena Kulelek, and Maban in Ethiopia.

In Ethiopia, it was determined that 12 group activities (each including 6–8 girls) would be administered across the camps, and in DRC, that 16 groups (each including 5–6 girls) would be administered across seven communities. In total, 165 adolescent girls participated in 28 groups across the two countries. Purposive sampling was used to ensure a maximum diversity of education levels, ages, and ethnicities were represented from the full study sample. The one exception was in Ethiopia, where participants lacking verbal fluency in Funj or Regarig were excluded due to the research team’s inability to locate literate female interviewers who spoke Maban and Ingessena Kulelek.

Quantitative data for these 165 adolescents was extracted from the larger dataset of 1788 adolescents who had completed the baseline survey. We were thus able to compare the quantitative and qualitative data for the 165 girls in the sub-sample. Pearson chi-square tests comparing demographic data found that in Ethiopia, adolescents in the sub-sample were slightly older than those in the full study population (mean difference 0.78, p < 0.001). Other demographic differences between the sub-sample of 165 and full study population in DRC and Ethiopia were not significant at an alpha of 0.05. Since the population chosen for the larger study drew from conflict-affected adolescents living in DRC and camp-dwelling adolescent refugees living in Ethiopia, the findings from this study are hypothesized to be generalizable to other adolescent girls living in these or similar communities. This paper compares the quantitative and qualitative results from this sub-sample of 165 adolescent girls.

### Instruments

#### Quantitative self-interview

The survey tool used in DRC and Ethiopia allowed for comparability across settings, and was field-tested for clarity and cultural relevance prior to baseline data collection. In the DRC, girls privately completed more sensitive questions on sexual health and violence using audio computer-assisted self-interviewing (ACASI). ACASI was selected based on previous evidence showing the administration format yielded significantly higher rates of disclosure on sensitive sexual topics among adolescent populations compared with face-to-face interviews [20–22]. Using ACASI, girls could simultaneously read the question in their primary language on the tablet, listen to the question through earphones, and select color-coded responses. Young adolescents, ages 10–12, completed a modified version of the survey that only included questions deemed appropriate through the formative work. In Ethiopia, because the survey was administered in non-written languages, the interview was administered entirely via ACASI, and study participants listened to questions that had been recorded in their primary language. There were no age-based adjustments to the survey in Ethiopia.

#### Qualitative group participatory activity

A qualitative participatory mapping activity was also developed and field-tested in each country prior to data collection to complement the survey to further understand experiences of GBV experienced by these adolescent populations. A trained local female facilitator invited a group of six to eight adolescents to draw a map of their community and to then identify safe and unsafe places using an unrestricted number of red and green stickers. Once the mapping was complete, the facilitator guided a discussion to better understand why participants had identified certain areas as safe or risky (unsafe), probe on violent experiences of in these spaces, and discuss support networks for adolescent girls who reported violence. Each discussion was recorded, transcribed, and translated for coding and analysis in English.

All caregivers were asked to provide informed consent for the girls’ participation in the study if the girl was under 18 years old and unmarried. Subsequently, girls were asked to assent for their participation in the study. Married girls and adolescents 18 or older were able to consent directly. In DRC, informed consent was read to potential participants through trained enumerators and written consent was obtained. In Ethiopia, informed consent was administered via audio recordings because the languages selected for the study are non-written; thus, informed consent forms were verbally translated into the appropriate languages and recorded. All potential participants listened to the same audio recording to ensure consistency in the informed consenting process and provided verbal consent. Data collection staff were trained and available to respond to any questions on the consenting process. Since languages were non-written, only verbal consent/assent was required for participants in Ethiopia.

All study procedures were approved by the Columbia University Institutional Review Board (IRB) and by in-country local bodies: the Ministry of Gender in DRC and the Administration for Refugee and Returnee Affairs in Ethiopia. (For additional details on the full evaluation study design, see Falb et al., 2016 [23]).

### Measures and codes

#### Quantitative measures

To assess experiences of violence, adolescents were asked whether they had been hit or beaten (physical violence), and whether they had been screamed at loudly or aggressively (emotional abuse). Sexual abuse was assessed by asking about unwilling sex, whether a respondent had been sexually coerced by others (based on influence or authority), and whether a respondent had experienced unwanted sexual touching. All of the variables were binary (yes/no). Girls ages 10–12 in the DRC were not asked about unwilling sex. Adolescents were also asked to identify perpetrators of physical violence, emotional abuse, and unwanted sexual touching.

#### Qualitative codes

To assess parallel outcome measures to the survey, codes included “physical violence”, “sexual violence” and “verbal abuse” and were utilized to analyze the emerging themes from the group discussions with study participants. Secondary coding explored perpetrators of this violence, and included “family members”, “intimate partners”, “soldiers or police”, “strangers”, and “other community members”. Additionally, location codes including “home”, “legal/military” (including police stations, soldiers’ camps), “NGO facilities” (including safe spaces and sports fields constructed by non-governmental organizations, or NGOs), “public areas” (including schools and health clinics), “natural environment” (including fields, forests, rivers, roads), and “water/sanitation” (including latrines, water points).

### Data analysis

Descriptive statistics and tests of variables of interest (chi-square tests) were analyzed using STATA 13.1. Qualitative data were analyzed using thematic content analysis [24]. Emerging themes were identified as central categories, and were used to identify recurring patterns in the data. Two members of the research team coded subsets of transcripts from both countries to ensure inter-coder reliability before coding the full data set. Narrative data from the group discussions were analyzed in NVivo 10.1.

## Results

### Survey results

Overall, 91.1% of the sample was living with at least one biological parent, 72.7% had ever attended school, and 25.5% had ever worked for money or payment (see Table 1). The mean age of the girls in the sample was 13.6 years (*SD* = 2.25 years): 12.0 years (*SD* = 1.5 years) in DRC, and 15.3 years (*SD* = 1.6 years) in Ethiopia. On average, adolescents in the sample had completed 3.2 years of school (*SD* = 1.7 years), with an average of 3.5 years (*SD* = 1.7 years) in DRC, and 2.7 years (*SD* = 1.5 years) in Ethiopia.

**Table 1:** Demographics of study population.

	Total		DRC		Ethiopia	
	(N = 165)		(N = 87)		(N = 78)	
	(n)	(%)	(n)	(%)	(n)	(%)
Living with a biological parent (%)						
Living with both parents	94	57.0	57	65.5	37	47.4
Living with mother only	47	28.5	25	28.7	22	28.2
Living with father only	11	6.7	1	1.2	10	12.8
Living with neither parent	8	4.9	4	4.6	4	5.1
Marital status^a^						
Unmarried	64	56.1	23	63.9	41	52.6
Married and living with partner	20	17.5	6	16.7	14	18.0
Married and not living with partner	14	12.3	3	8.3	11	14.1
Living with partner as if married	6	5.3	2	5.6	4	5.1
Education						
Ever attended school	120	72.7	71	81.6	49	62.8
Enrolled in school in last school year^b^	81	67.5	44	62.0	37	75.5
Reasons for not being enrolled in school (%)	(N = 39)		(N = 27)		(N = 12)	
Family could not afford	26	66.7	25	92.6	1	8.3
Got pregnant or married	2	5.1	0	0.0	2	16.7
Too many domestic responsibilities	4	10.3	0	0.0	4	33.3
School too far/no school in vicinity	0	0.0	0	0.0	0	0.0
Family does not approve/see benefit	0	0.0	0	0.0	0	0.0
Other	3	7.7	2	7.4	1	8.3
Did not know or no response	4	10.3	0	0.0	4	33.3

^a^ Numbers reported represent the total sample who were asked about marital status (N = 114; N = 36 in DRC, N = 78 in Ethiopia). Girls age 10–12 in DRC were not asked about marital status (N = 51).

^b^ Percentage listed is of the total who had ever attended school.

Table 2 shows the prevalence of reported experiences of physical, emotional, or sexual violence, and perpetrators of that violence. Eighty-seven (52.7%) adolescents reported experiencing at least one form of physical, emotional, and/or sexual violence.

**Table 2:** Prevalence and perpetrators of past year violence^a^, reported via ACASI.

	Total		DRC		Ethiopia	
	(N = 165)		(N = 87)		(N = 78)	
Prevalence of Violence						
	(n)	(%)	(n)	(%)	(n)	(%)
Physical violence						
Beaten or hit	51	30.9	34	39.1	17	21.8
Emotional abuse						
Screamed at loudly or aggressively	55	33.3	36	41.4	19	24.4
Sexual abuse						
Unwanted sexual touching	23	15.2	13	16.1	10	14.3
Sexual coercion	20	13.3	12	14.8	8	11.4
	(N = 104)		(N = 33)		(N = 71)	
Forced sex	12	11.7	7	21.2	5	7.1
Perpetrators of Violence						
Physical violence	(N = 51)		(N = 34)		(N = 17)	
	(n)	(%)	(n)	(%)	(n)	(%)
Boyfriend or husband	17	33.3	14	41.2	3	17.7
Parent, caregiver, or other relative	14	27.5	8	23.5	6	35.3
Friend or neighbor	9	17.7	7	20.6	2	11.8
Member of an armed group	3	5.9	1	2.9	2	11.8
Official (police, teacher, religious or local leader)	4	7.8	2	5.9	2	11.8
Other	5	9.8	3	8.8	2	11.8
Emotional abuse—loud or aggressive screaming	(N = 57)		(N = 36)		(N = 19)	
	(n)	(%)	(n)	(%)	(n)	(%)
Boyfriend or husband	18	31.6	14	38.9	4	21.1
Parent, caregiver, or other relative	25	43.9	14	38.9	11	57.9
Friend or neighbor	8	14.0	6	16.7	2	10.5
Member of an armed group	1	1.8	0	0.0	1	5.3
Official (police, teacher, religious or local leader)	1	1.8	0	0.0	1	5.3
Other	4	7.0	2	5.6	2	10.5
Unwanted sexual touching	(N = 23)		(N = 13)		(N = 10)	
	(n)	(%)	(n)	(%)	(n)	(%)
Boyfriend or husband	11	47.8	9	69.2	2	20.0
Parent, caregiver, or other relative	6	26.1	1	7.7	5	50.0
Friend or neighbor	2	8.7	1	7.7	1	10.0
Member of an armed group	0	0.0	0	0.0	0	0.0
Official (police, teacher, religious or local leader)	0	0.0	0	0.0	0	0.0
Other	4	17.4	2	15.4	2	20.0
Sexual coercion	(N = 20)		(N = 12)		(N = 8)	
	(n)	(%)	(n)	(%)	(n)	(%)
Boyfriend or husband	11	55.0	6	50.0	5	62.5
Parent, caregiver, or other relative	2	10.0	1	8.3	1	12.5
Friend or neighbor	4	20.0	2	16.7	2	25.0
Member of an armed group	1	5.0	1	8.3	0	0.0
Official (police, teacher, religious or local leader)	1	5.0	1	8.3	0	0.0
Other	2	10.0	2	16.7	0	0.0

^a^ Study participants were allowed to select more than one category of perpetrator for each form of violence, so N for perpetrator categories does not equal total N who reported experiencing that form of violence.

Intimate partners (boyfriends or husbands) or caregivers were identified as perpetrators of violence by 60.8% of adolescents who experienced physical violence, 76.4% who experienced emotional abuse, and 81.6% who experienced any form of sexual abuse. Overall, 77.0% (n = 67) of adolescents who reported any form of violence reported that an intimate partner or caregiver was a perpetrator of at least one form of violence. Additionally, 24.1% (n = 21) of adolescents reported perpetration from friends or neighbors, 6.9% (n = 6) from a member of an armed group, 9.2% (n = 8) from officials, and 18.39% (n = 16) from someone else, for at least one form of violence.

### Qualitative results

#### Types of violence and perpetrators

Study participants described three primary types of violence: emotional and verbal abuse, physical violence, and sexual violence. Overall, verbal abuse, often described as “fighting”, was most frequently discussed. Participants in both countries described how boys, drunk men, and other girls perpetrated this form of violence in public settings such as water points, sports fields, schools, markets, and roads. As one participant stated, “The red [sticker] is to show the conflict and fight among the different tribes in the market…there are a lot of drunk people. If a man tried to harass me verbally, I will get into conflict with him” *(age 15*, *Ethiopia)*. Participants also frequently noted verbal arguments at the sports fields:

“We put red [stickers] there because sometimes the boys disturb us as they want to play with us by force. When it is boy’s day, we don’t go there. But when it is our day, boys come and fight with us…they insult us and they also beat us”

(age 14, Ethiopia)

Physical violence was often described in both countries as a consequence of escalated verbal abuse, or in relation to tasks undertaken in the forest, at the river, or on the roads between these other locations. For example, in discussing the dangers of collecting firewood in the forest, one participant shared, “some men hide themselves in the trees and suddenly come and beat us” *(age 14*, *Ethiopia)*. Another participant noted, “drunk people go [to the river] at 10 a.m. and they beat girls and that may even lead to death” *(age 15*, *Ethiopia)*. In the DRC, girls described physical violence as often being perpetrated by military or police personnel. One participant noted, “Military soldiers …if you come at night alone they can catch, beat and maybe shoot you” (*age 13*, *DRC)* Another shared, “At a soldiers’ camp they beat people, hurt them; it is not peaceful there” *(ages 10–14*, *DRC)*. In Ethiopia, there was no mention of soldiers or police personnel in relation to physical violence. Rather, participants tended to generalize perpetrators of physical violence to strangers, ‘other refugees’ and host community members.

In some groups, adolescents referenced physical violence between family members. Participants shared that girls of all ages suffer such violence when they do something perceived as ‘wrong’ such as going somewhere without parental permission. As one participant noted, “when [girls] want to go to somewhere and parents forbid them, they go without permission. So they are beaten”. This same participant continued, “girls who live with their parents are beaten, but those who live without parents are free and no one beats them; they lead their own lives”(*age 17*, *Ethiopia)*.

Sexual violence was mentioned in all discussion groups in Ethiopia and in a majority of the groups in the DRC. Despite the fact that sexual violence was cited as the most serious type of violence affecting the community, references to perpetrators were often vague. For example, one participant described, “sometimes there are drunk men who rape girls along the road” (*age 15*, *Ethiopia)*. Another adolescent shared, “Sometimes when we go to the forest to collect firewood, the local people chase us out of the forest. If we don’t meet the local people, it is safe…There is also rape” (*age 15*, *Ethiopia*). Residing with a husband or one’s parents was identified as protective factors against rape in Ethiopia. As one adolescent stated, “Married girls have husbands who the perpetrators are afraid of. But unmarried girls have no one who can protect them” (*age 15*, *Ethiopia*). Another shared that rape happens “to girls who live without parents” (*age 16*, *Ethiopia*). Overall, participants rarely identified perpetrators as known to them, instead primarily describing violence as committed by strangers.

#### Location

The most frequently identified safe spaces across both countries were homes, religious spaces (churches and mosques), health centers or hospitals, roads, and schools. The most frequently identified unsafe spaces were forests, roads, schools, markets, and sports fields.

Interestingly, homes—the most frequently identified ‘safe location’ across both countries—were co-identified as safe and unsafe in some groups, primarily in one camp in Ethiopia. In examining the transcripts, however, we found that adolescents provided limited or no response as to what made a home unsafe, even after a facilitator probed multiple times. On the few occasions where participants were willing to discuss homes as unsafe, adolescents discussed rape perpetrated by strangers. As one participant described, “[rape] can happen that you spend night alone in house; a bandit can come there, strangles you or rapes you” *(ages 10–14*, *DRC)*. Another participant stated, “I live with my family, but in a separate house. If I forget to close my door and go to sleep, I might be raped…by strangers” *(age 18*, *Ethiopia)*. Of the select participants who mentioned rape in their home, they tended to refer to separate living quarters within the family compound, and “strangers” or “bandits” as the perpetrators.

## Discussion

These findings surprised our local and international research teams. The two approaches had been designed to elicit complementary data on experiences of violence among adolescents. Instead, the two methods show strikingly different pictures of adolescent exposure to violence. Participatory group discussions primarily focused on public spaces as unsafe, and perpetrators as strangers and community members. Very little discussion included mention of family or intimate partner violence (IPV), aside from references to some harsh ‘disciplinary’ action of caregivers. This articulation of girls’ experiences of violence stands is in stark contrast to the quantitative findings, conducted confidentially with ACASI, which suggested that the majority of physical, emotional and sexual violence is being perpetrated by boyfriends, husbands and caregivers. For example, 26 of the 36 adolescents who reported unwanted sexual touching in the survey named an intimate partner or caregiver as the perpetrator, yet there was no mention of these people as perpetrators in group activities. The quantitative findings are consistent with adult women’s reporting in humanitarian settings in that violence in the home (e.g., IPV) often occurs at higher frequency and is of greater concern than other forms of non-partner perpetrated violence [25, 26].

The differences seen in the data are likely attributable to a few factors. First, the line of inquiry around experiences of violence followed best practices for surveys and group discussions, but likely encouraged the elicitation of different information. “Gate questions”—which tend to be broad and attempt to stimulate recall of many potentially relevant events through a single question—have been shown to elicit lower prevalence rates of violence when compared with a series of behavioral- and relationship-specific questions [27, 28]. Broader gate questions, such as “*In what ways are you and other girls unsafe in this location*?” are appropriately employed in groups in an attempt to let participants guide the direction of the discussion. It is likely that asking these broader gate questions may shape the types of violent events that adolescents recall and deem relevant for discussion in front of others. It is possible that a broad gate question, even with significant follow-up probing, might not have triggered recall of domestic or intimate partner violence among our study population in the group discussions.

In contrast, employing a series of behavioral and relationship-specific questions—as was done in the ACASI survey—can specifically trigger recall events of interest. By asking multiple questions, such as “*Has anyone ever hit or beat you and hurt your body*?” followed by “*Who has hurt you in this way*?*”* the respondent is provided time and space to think about different categories of violence perpetrated by different people in their lives. Yet, these differing approaches to questioning may not alone sufficiently explain the discrepancy in our results.

Beyond the different lines of questioning, other forces likely shaped the narratives emerging from the group- versus individual-level data. Existing community norms around ‘acceptable’ types of violence for public or group discussions may have influenced adolescents in their discussions in both countries. Previous research has explored IPV as a learned social behavior, with many cultures condoning the use of violence by men against women in certain circumstances and within certain boundaries of severity [29]. Additionally, cross-cultural studies suggest IPV is more prevalent in societies where overall violence is prevalent, including conflict-affected settings [30].

Often unspoken, these norms suggest societal expectations of appropriate and inappropriate behavior, governing what is (and is not) acceptable to discuss and influencing interactions with others [31]. Such norms are reinforced by social narratives and frames [32, 33], which “shape our views on what counts as a problem…and what does not… which events will be noticed…and which will not…” [34].

Both internal and external pressures are understood to maintain community norms and social narratives of violence, particularly violence against women and girls [31, 35]. Within families and communities, individuals may be discouraged from discussing abuse by intimate partners or caregivers because of threat of social disapproval or feelings of guilt and shame that result from the internalization of cultural norms [31, 36]. Adolescent girls may be particularly reticent to discuss violence by caregivers or intimate partners in group settings, due to an internalization of victim-blaming norms and continuing reliance on these perpetrators to meet their basic needs.

At a societal level, laws and policies can assist in maintaining or discouraging norms linked to violence. Sudan offers no protection in the law for marital rape [37]. Similarly, there is no mention of domestic violence in the DRC's Penal Code [38], or its Family Code [39]. Without explicit recognition of IPV or domestic violence in national legislation, there is little recourse for survivors [40]. These omissions diminish recognition of certain experiences of violence.

Finally, the international community, including humanitarian actors and the media, has likely played a role in shaping social narratives and reinforcing community norms around violence. Autesserre (2012) has argued that a simple story line building on narratives already familiar to the public and offering a comparatively simple solution enables a social narrative to achieve dominance [34]. For the past few decades, advocacy campaign messages have focused on rape perpetrated by strangers and rebel groups, for example, while fetching water or collecting firewood. Mirroring these messages, interventions have focused on increasing safety in public spaces through lit pathways, gender-separate latrines, or fuel alternatives to limit firewood collection in unsafe areas. These interventions, certainly important in their own right, reinforce a simple, solution-oriented narrative of stranger violence and violence in public spaces that dominated the group discussions in this study.

### Limitations

This study is not without limitations. While our protocols followed standard practice for probing on the same research question in a survey versus group-based discussion, this means that the same probes were not systematically used in both methods. One might argue that a comparison between results is difficult given that the group discussions did not follow the exact same question format as the survey asking explicitly about physical, emotional and sexual violence, and followed by specific questions about perpetrators. We acknowledge this as a limitation, yet still believe our analysis has merit given the fact that group discussions tend to follow a more open structure compared to surveys. In addition, our study did not compare differences between ACASI and face-to-face interviews. The extant research comparing ACASI and face-to-face interviews suggests that our use of ACASI may have fostered disclosures around IPV and familial violence beyond what might have been revealed through individual face-to-face interviews, had we also included face-to-face interviews as part of our protocol. This question could benefit from additional research.

## Conclusion

More valid measures can help researchers and practitioners to fill knowledge gaps and provide a broader understanding of how IPV and abuse from caregivers fits into a broader, politically violent landscape. Preliminary research, for example, has begun to provide evidence that family violence—more so than political violence—is a consistent predictor of youth mental health trajectory [41], and that political conflict that separates households, disrupts family access to economic resources and social support, can exacerbate the perpetration of GBV in the household [42]. These findings, in conjunction with our own, highlight the need to conceptualize GBV in conflict settings not merely as centered in the political or public sphere, but also as situated within the home and within intimate partnerships. Traditional conceptualizations of violence in conflict that ignore more intimate forms of violence risk impeding program design and effectiveness, and continuing to perpetuate simplistic narratives of GBV in conflict.

## Supporting information

S1 File COMPASS baseline survey, English. (PDF)
