# Supplementary material for: Disclosure bias for group versus individual reporting of violence amongst conflict-affected adolescent girls in DRC and Ethiopia
Source: PLoS One. 2017 Apr 4;12(4):e0174741. doi: 10.1371/journal.pone.0174741 (PMC5380345; doi:10.1371/journal.pone.0174741)
Supplement: S1 File — (PDF) [file pone.0174741.s001.pdf]

Supporting Information

Below is the English version of the COMPASS baseline survey administered to adolescent girl participants. The Funj, Regarig, Engesena Quickly, and Maban (non-written languages) versions of the survey is only available as audio files.

**fGirl survey (ETH)**			
**Question #**	**Question**	**Response options**	**Instructions**
**A. Administrative Questions**	**INTERVIEWER: ANSWER THE FOLLOWING QUESTIONS ON THE ACASI TOOL BEFORE THE INTERVIEW**		
A1	Name of camp	_____ _____ _____	Record
A2	Zone	A<br>B<br>C<br>D<br>E<br>F<br>G<br>H<br>I<br>J	Record from listing
A4	Surveyor ID	_____ _____	Record your ID here
A5	Date of survey	____ ____/____ ____/____ ____ ____ ____	dd/mm/yyyyy
A6	Start time of survey	_____ _____ : _____ _____	24 hour clock
A7	End time of survey	_____ _____ : _____ _____	24 hour clock
A10	Girl's Identification Code	xxx-xxx-xxx	
**B. Demographics**	**Welcome to using the ACASI tablet. My name is _______ and I am going to read you the questions. Remember that you can call the interviewer over any time you have questions or problems with the computer. We are going to start with some easy questions about you.**		
B1	How old are you?	1= 9 or younger<br>2= 10-12<br>3= 13-14<br>4=15-17<br>5=18-19<br>6=20 or older<br>888=Don't Know<br>999 = No Response	If response is less than age 13 or don't know, end the survey.
B2	Have you ever attended school?	1=Yes<br>2=No<br>888=Don't Know	If the respondent answers no or don't

fGirl survey (ETH)			
**Question #**	**Question**	**Response options**	**Instructions**
		999 = No Response	know, skip to question B8
B3	What was the highest grade you completed in another country (Sudan or South Sudan)?	_____ _____<br><br>888=Don't Know<br><br>999=No Response	COUNTER GRAPHIC<br><br>Range 1-13
B3a	What was the highest grade you completed in Ethiopia?	_____ _____<br><br>888=Don't Know<br><br>999=No Response	COUNTER GRAPHIC<br><br>Range 1-13
B4	Were you enrolled in school during the most recent school year (2014-2015)?	1=Yes<br>2=No<br>888=Don't Know<br>999 = No Response	If "Yes", skip to question B6<br><br>If not "Yes" ask question B5
B5	What is the primary reason you were not enrolled in school during the most recent school year (2014-2015)?	1=Family could not afford<br>2=Got pregnant or married<br>3=Too many domestic responsibilities<br>4=School too far / no school in vicinity<br>5=Family does not approve/see benefit<br>6=Other<br>888=Don't Know<br>999=No Response	Skip to question B8
B8	Aside from your regular household chores, have you ever worked for money or any other payment?	1=Yes<br>2=No<br>888=Don't Know<br>999 = No Response	If answer is "No" or don't know", skip to question B10
B9	What type of work have you done in the past year for money or any other payment?	1=Housekeeping or childcare for another family<br>2=Farm work<br>3=Handicrafts<br>4=Collecting firewood<br>5=Small business, such as selling tea or cookies, shop work<br>6=Other<br>888=Don't Know<br>999=No Response	Check all that apply

fGirl survey (ETH)			
**Question #**	**Question**	**Response options**	**Instructions**
B10	Aside from your regular household chores, what kinds of work have you done in the past year for which you were not paid?	1=Housekeeping or childcare for another family<br>2=Farm work<br>3=Handicrafts<br>4=Collecting firewood<br>5=Small business, such as selling tea or cookies, shop work<br>6=Other<br>888=Don't Know<br>999=No Response	Check all that apply
B11	Now, I would like to ask you some questions about your biological parents, your natural parents who gave birth to you. Which of your biological parents is living with you?	1=Father<br>2=Mother<br>3=Both<br>4=Neither<br>888=DK<br>999=NR	
**C. Relationships with adults**	**You are doing a great job!**		
C1	There is an adult in your life who provides guidance, moral support, listens to you, and gives you good advice to help you succeed in life. Would you say this is…	3=Very true<br>2=Somewhat true<br>1=Not true at all<br>888=Don't Know<br>999=No Response	
C3	Who is this person?	1=Mother<br>2=Father<br>3=Other relative (sister/brother/Grandmother/Grandfather/Aunt/Uncle)<br>4= Boyfriend/husband<br>5=Other adult<br>6=Girl program leader<br>888=Don't Know<br>999=No Response	
C4	What do you talk to this person about?	1=Plans for future (school, work or career, marriage)<br>2=Health or school issues<br>3=Conflict with your parents or guardians<br>4=Conflicts with husband, men, or boyfriends<br>5=Conflicts with friends, neighbors, or siblings<br>6=Financial plans/saving money<br>888=Don't Know<br>999=No Response	Check all mentioned.

**fGirl survey (ETH)**			
**Question #**	**Question**	**Response options**	**Instructions**
	**Next I am going to ask you how comfortable are you talking about a few specific issues with your parent(s)/caregiver(s). For each topic I mention, I want you to choose whether you are very comfortable or uncomfortable talking about it with these people. It is alright if you don't know or do not want to answer.**		
C5a	Are you uncomfortable or comfortable talking about your education with your parent(s)/caregiver(s)?	1=Comfortable<br>2=Uncomfortable<br>888=Don't Know<br>999=No Response	
C5b	What about what you will do to earn a living in the future?	1=Comfortable<br>2=Uncomfortable<br>888=Don't Know<br>999=No Response	
C5c	What about marriage and when your parents /caregivers expect you to get married?	1=Comfortable<br>2=Uncomfortable<br>888=Don't Know<br>999=No Response	
C5d	What about puberty (changes that happen to kids as they grow up)?	1=Comfortable<br>2=Uncomfortable<br>888=Don't Know<br>999=No Response	
C5e	Are you uncomfortable or comfortable talking about topics related to sex matters with your parent(s)/caregiver(s)?	1=Comfortable<br>2=Uncomfortable<br>888=Don't Know<br>999=No Response	
C5f	What about how to avoid getting pregnant?	1=Comfortable<br>2=Uncomfortable<br>888=Don't Know<br>999=No Response	

fGirl survey (ETH)			
**Question #**	**Question**	**Response options**	**Instructions**
C5g	What about HIV/AIDS or other sexually transmitted diseases (STDs)?	1=Comfortable<br>2=Uncomfortable<br>888=Don't Know<br>999=No Response	
**D. Topics related to safety**	**Great work so far! The next questions relate to how you feel about safety in your life and community and places you feel safe.**		
D1	Do you feel safe at home?	1=Yes<br>2=No<br>888=Don't Know<br>999 = No Response	
D2	Do you feel safe at school?	1=Yes<br>2=No<br>888=Don't Know<br>999 = No Response	If answered 2=No to B2, skip this question
D3	Do you feel safe at a friend's house?	1=Yes<br>2=No<br>888=Don't Know<br>999 = No Response	
D4	Do you feel safe at a neighbour's house?	1=Yes<br>2=No<br>888=Don't Know<br>999 = No Response	
D5	Is there a place in the community where you feel comfortable spending time with other girls?	1=Yes<br>2=No<br>888=Don't Know<br>999 = No Response	
	**Next I'd like to ask you a couple questions about your friends. It is alright if you don't know or do not want to answer.**		
D8	Do you have any female friends your own age outside of the family?	1=Yes<br>2=No<br>888=Don't Know<br>999 = No Response	If "No" or "don't know, skip to question D10
D9	How many of these female friends do you have?	0=0<br>1=1 to 3<br>2=4 to 10<br>3=More than 10<br>888=Don't Know<br>999=No Response	

**fGirl survey (ETH)**			
**Question #**	**Question**	**Response options**	**Instructions**
D10	Are there any women outside of your family who you can go to with problems?	1=Yes<br>2=No<br>888=Don't Know<br>999 = No Response	
**E. Aspirational attitudes**	**Now I am going to ask you a couple of questions about things that happen to a girl throughout her life, like school and marriage.**		
E1	What grade in school do you think a girl should complete before leaving school?	_____ _____ (grade level)<br><br>888=Don't Know<br><br>999=No Response	COUNTER GRAPHIC<br><br>Range 1-13
E2	At what age do think a girl should get married?	1=<10 years old<br>2=10-14 years old<br>3=15-17 years old<br>4=18-20 years old<br>5=>20 years old<br>888=Don't Know<br>999=No Response	Enter 88 for Don't Know<br><br>Enter 99 for No Response
E3	At what age do you think a girl should have her first child?	1=<10 years old<br>2=10-14 years old<br>3=15-17 years old<br>4=18-20 years old<br>5=>20 years old<br>888=Don't Know<br>999=No Response	Enter 88 for Don't Know<br><br>Enter 99 for No Response
E4	Do you think it is okay for a girl to try to earn money or have a job outside of the home, even after marriage?	1=Yes<br>2=No<br>888=Don't Know<br>999=No Response	
**F. Gender Relations**			
	**Now, I am going to read some statements about relationships between men and women. For each statement I read, I want you to say whether you agree or disagree. There are no right or wrong answers, so please feel free to share your honest opinion. I would like to know *your* opinion about these statements. It is alright if you do not know or do not want to answer.**		
F1	It is a female's responsibility to avoid getting pregnant.	1=Agree<br>2=Disagree<br>888=Don't Know<br>999=No Response	
F2	A male should have the final word about decisions in his home.	1=Agree<br>2=Disagree<br>888=Don't Know<br>999=No Response	

fGirl survey (ETH)			
**Question #**	**Question**	**Response options**	**Instructions**
F3	A female should tolerate violence to keep the family together.	1=Agree<br>2=Disagree<br>888=Don't Know<br>999=No Response	
F4	It is OK for a male to hit his wife if she will not have sex with him.	1=Agree<br>2=Disagree<br>888=Don't Know<br>999=No Response	
F5	Males and females should share household chores.	1=Agree<br>2=Disagree<br>888=Don't Know<br>999=No Response	
**G. Self-esteem**	**I am going to read you 10 statements that could possibly apply to you. Please select how much you agree with each statement: whether you strongly agree, agree, disagree or strongly disagree. It is alright if you don't know or if you do not want to answer.**		
G1	I feel that I am equal to other people.	4= Strongly agree<br>3=Agree<br>2=Disagree<br>1=Strongly disagree<br>888=Don't Know<br>999=No Response	
G2	I feel that I have a number of good qualities.	4= Strongly agree<br>3=Agree<br>2=Disagree<br>1=Strongly disagree<br>888=Don't Know<br>999=No Response	
G3	I feel that I am a failure.	1= Strongly agree<br>2=Agree<br>3=Disagree<br>4=Strongly disagree<br>888=Don't Know<br>999=No Response	
G4	I am able to do things as well as most other people.	4= Strongly agree<br>3=Agree<br>2=Disagree<br>1=Strongly disagree<br>888=Don't Know<br>999=No Response	
G5	I feel I have much to be proud of.	4= Strongly agree<br>3=Agree<br>2=Disagree<br>1=Strongly disagree<br>888=Don't Know<br>999=No Response	

fGirl survey (ETH)			
**Question #**	**Question**	**Response options**	**Instructions**
G6	I take a positive attitude toward myself.	4= Strongly agree<br>3=Agree<br>2=Disagree<br>1=Strongly disagree<br>888=Don't Know<br>999=No Response	
G7	I am satisfied with myself.	4= Strongly agree<br>3=Agree<br>2=Disagree<br>1=Strongly disagree<br>888=Don't Know<br>999=No Response	
G8	I have a lot of respect for myself.	4= Strongly agree<br>3=Agree<br>2=Disagree<br>1=Strongly disagree<br>888=Don't Know<br>999=No Response	
G9	I feel that I am a useful person.	4= Strongly agree<br>3=Agree<br>2=Disagree<br>1=Strongly disagree<br>888=Don't Know<br>999=No Response	
G10	I think that I am a person who has value	4= Strongly agree<br>3=Agree<br>2=Disagree<br>1=Strongly disagree<br>888=Don't Know<br>999=No Response	
**H. Sexual History**	**Now I want to ask you some questions about your own experience in relationships with boys and men. Please remember that there are no right or wrong answers and be assured that you will not be judged in any way.**<br><br>**Also recall that your answers are completely anonymous (your name is nowhere on this survey), as well as confidential, meaning that no one outside of the research team will know your answers. Since you are using this tablet, not even the interviewer that you talked to before will know how you answered. You can also say you don't know or refuse to answer any question and nothing bad will happen to you.**		
H1	Are you currently married or living with someone as if married?	1=Not married and not living with someone as if married<br>2=Yes, married and living with husband<br>3=Yes married but not living with husband<br>4=Living with a man as if married<br>888=Don't Know	If "No", "Don't Know", or "No Response", skip to H3

fGirl survey (ETH)			
**Question #**	**Question**	**Response options**	**Instructions**
		999=No Response	
H2	How old is your husband?	1= 10-17<br>2= 18-29<br>3=30-49<br>4=50 or older<br>888=Don't Know<br>999=No Response	
H3	At what age do you think a girl is old enough to have a boyfriend?	1= 5 or younger<br>2= 6-9<br>3=10-13<br>4=14-17<br>5=18 or older<br>888=Don't Know<br>999=No Response	
H4	At what age do you think a girl is old enough to choose to have sex?	1= 5 or younger<br>2=6-9<br>3=10-13<br>4=14-17<br>5=18 or older<br>888=Don't Know<br>999=No Response	
H8	Have you ever had a boyfriend?	1=Yes<br>2=No<br>888=Don't Know<br>999=No Response	If the answer is "No" or "don't know", skip to H10
H10	Have you ever had sexual intercourse willingly or unwillingly?	1=Yes<br>2=No<br>888=Don't Know<br>999=No Response	If the answer is not "No" or "Don't know", skip to question H16
H11	How old were you when you willingly had sex the first time?	1= 5 or younger<br>2=6-9<br>3=10-13<br>4=14-17<br>5=18 or older<br>777=Never had sex willingly<br>888=Don't Know<br>999=No Response<br>888=Don't know<br>999=No Response	If answer is "Never" skip to question H14
H12	When was the LAST time you willingly had sex?	1= Less than one week ago<br>2=Between 1 week and 1 month ago<br>3=Between 1 month and 3 months ago<br>4=Between 3 months and 1 year ago<br>5=More than a year ago<br>888=Don't Know<br>999=No Response	

fGirl survey (ETH)			
**Question #**	**Question**	**Response options**	**Instructions**
H13	What type of protection did you use the LAST time you willingly had sex??	1=Condoms<br>2=Withdrawal<br>3=Birth control pills or injection to avoid pregnancy<br>4=Other<br>5=None<br>888=Don't Know<br>999=No Response	Check ALL that apply
H14	How old were you when you unwillingly had sex for the first time?	1= 5 or younger<br>2=6-9<br>3=10-13<br>4=14-17<br>5=18 or older<br>777=Never had sex unwillingly<br>888=Don't Know<br>999=No Response	If answer is "Never" skip to question H16
H15	When was the LAST time you unwillingly had sex?	1= Less than one week ago<br>2=Between 1 week and 1 month ago<br>3=Between 1 month and 3 months ago<br>4=Between 3 months and 1 year ago<br>5=More than a year ago<br>888=Don't Know<br>999=No Response	
H16	Of your closest friends, how many do you think have ever willingly had sex?	1=None of my friends<br>2=Some of my friends<br>3=Most of my friends<br>4=All of my friends<br>888=Don't Know<br>999=No Response	
H17	Of your closest friends, how many do you think have ever unwillingly had sex?	1=None of my friends<br>2=Some of my friends<br>3=Most of my friends<br>4=All of my friends<br>888=Don't Know<br>999=No Response	
**I. Domestic Violence Attitudes**	**Sometimes a husband is annoyed or angered by things that his wife does. I am going to read a few situations. For each one, I want you to say whether you think a husband is justified in hitting or beating his wife. Remember, there are no right or wrong answers and you can say you don't know or that you do not want to answer each question.**		
I1	Is it OK for a husband to beat or hit his wife if she goes out without telling him?	1=Yes<br>2=No<br>888=Don't Know<br>999=No Response	

fGirl survey (ETH)			
**Question #**	**Question**	**Response options**	**Instructions**
I2	Is it OK for a husband to beat or hit his wife if she does not care for her children in the proper way?	1=Yes<br>2=No<br>888=Don't Know<br>999=No Response	
I3	Is it OK for a husband to beat or hit his wife if she argues with him?	1=Yes<br>2=No<br>888=Don't Know<br>999=No Response	
I4	Is it OK for a husband to beat or hit his wife if she refuses to have sex with him?	1=Yes<br>2=No<br>888=Don't Know<br>999=No Response	
I5	Is it OK for a husband to beat or hit his wife if she burns the food?	1=Yes<br>2=No<br>888=Don't Know<br>999=No Response	
**J. Physical Violence and emotional and verbal abuse**	**The next set of questions is about other things that may have happened to you. We understand this may make you feel bad to think about, but we hope that you will share some information with us so we can better understand the needs and concerns of girls like yourself in Ethiopia. Some of these questions are personal but keep in mind that your name is not on the survey and because you are using this computer, no one else will know your answers. Please remember that anything that has been done to you is not your fault. Please feel free to answer openly and honestly. You can skip any question that you don't feel comfortable answering.**		
J1	In the past 12 months, has anyone ever hit or beat you and hurt your body?	1=Yes<br>2=No<br>888=Don't Know<br>999=No Response	If "No" or "Don't know" or "no response" skip to J3
J2	Who has hurt you in this way?	1=Boyfriend or husband<br>2=Parent, caregiver or other relative<br>3=Friend or neighbour<br>4= Member of an armed group<br>5= Official<br>6=Other<br>999=No Response	Check all that apply
J3	In the past 12 months, has anyone ever screamed at you loudly or aggressively?	1=Yes<br>2=No<br>888=Don't Know<br>999=No Response	If "No" or "Don't know" or "no response" skip to J7
J4	Who has screamed at you loudly or aggressively?	1=Boyfriend or husband<br>2=Parent, caregiver or other relative<br>3=Friend or neighbour<br>4= Member of an armed group<br>5= Official<br>6=Other<br>999=No Response	Check all that apply

fGirl survey (ETH)			
**Question #**	**Question**	**Response options**	**Instructions**
J7	In the past 12 months, have you felt that you are not cared for by the person who should care for you?	1=Yes<br>2=No<br>888=Don't Know<br>999=No Response	If "No" or "Don't know" or "no response" skip to section K
J8	In the past 12 months, how often have you felt that you are not cared for?	1=Many times<br>2=Sometimes<br>3=Never<br>888=Don't Know<br>999=No Response	
**K. Sexual Violence**	**Thank you for answering those questions about things you have experienced. I know answering this sort of question is not easy. The next section also has some difficult questions, but we hope that by using this computer you feel comfortable to tell us what you have experienced.**<br><br>**Girls and women may experience unwanted sexual contact by people they know well, such as a romantic partner, family member or friend, or by strangers. The questions in these sections are personal, and may be uncomfortable to answer, but you will help us understand people's experience of sexual contact without their permission. Your answers are confidential and you can skip any questions you prefer not to answer. Also remember that you will not be judged and that there are no right or wrong answers and that anything that has been done to you without your explicit permission or done by an adult is not your fault.**		
K1	Has anyone ever touched you in a sexual way without your permission? By touched in a sexual way I mean pinching, grabbing, or fondling.	1=Yes<br>2=No<br>888=Don't Know<br>999=No Response	If not "Yes" skip to question K4
K2	Who was the person who did this?	1=Boyfriend or husband<br>2=Parent, caregiver or other relative<br>3=Friend or neighbour<br>4= Member of an armed group<br>5= Official<br>6=Other<br>999=No Response	Check all that apply
K3a	In the past 12 months, has anyone touched you in a sexual way without your permission?	1=Yes<br>2=No<br>888=Don't Know<br>999=No Response	If "No" or "Don't know" skip to question K4
K3	How many times in the past 12 months has someone touched you in a sexual way without your permission?	_____ _____<br><br>888=Don't Know<br><br>999=No Response	COUNTER GRAPHIC
K4	Has anyone ever tried to use their influence or authority to threaten or pressure you to have sex? For example, saying they will give you bad grades or do some bad thing if you do not have sex with them?	1=Yes<br>2=No<br>888=Don't Know<br>999=No Response	If no or don't know skip to question K6a

fGirl survey (ETH)			
**Question #**	**Question**	**Response options**	**Instructions**
K5	Who was the person who did this?	1=Boyfriend or husband<br>2=Parent, caregiver or other relative<br>3=Friend or neighbour<br>4= Member of an armed group<br>5= Official<br>6=Other<br>999=No Response	Check all that apply
K6a	In the past 12 months, have you had sex with someone because they threatened you or pressured you by using their influence or authority?	1=Yes<br>2=No<br>888=Don't Know<br>999=No Response	If "No" or "Don't know" skip to question K9
K6	How many times in the past 12 months have you had sex with someone because they used words or threats to make you have sex?	_____ _____<br>888=Don't Know<br>999=No Response	COUNTER GRAPHIC
K9	Has anyone ever given you money, food, gifts or any favors to have sex with them?	1=Yes<br>2=No<br>888=Don't Know<br>999=No Response	If "No" or "Don't know" skip to question K11
K10	Has this occurred in the past 12 months?	1=Yes<br>2=No<br>888=Don't Know<br>999=No Response	
	**Please tell me if you agree or disagree with each of the following statements. Remember, there are no right or wrong answers and you can say you don't know or that you do not want to answer each question.**		
K11	My family would blame me if I was forced to have sex	1=Agree<br>2=Disagree<br>888=Don't Know<br>999=No Response	
K12	My community would force me to marry a man if he forced me to have sex	1=Agree<br>2=Disagree<br>888=Don't Know<br>999=No Response	
K13	I have someone in the community I would trust to talk to if I was forced to have sex	1=Agree<br>2=Disagree<br>888=Don't Know<br>999=No Response	
**L. Services**	**Thank you for answering those difficult questions, you did an excellent job. I promise that there will be no more questions like that about things you have experienced. Now I want to ask you some questions about services that may be available to girls like you.**		

fGirl survey (ETH)			
**Question #**	**Question**	**Response options**	**Instructions**
L1	If someone has sex with, or tries to have sex with, a girl when she does not want to, do you know of a place she can go for help?	1=Yes<br>2=No<br>888=Don't Know<br>999=No Response	
L2	Do you know of a place a girl could go for help if someone hit her?	1=Yes<br>2=No<br>888=Don't Know<br>999=No Response	
**O. Wrap Up Questions for Respondent**	**OK, thank you for answering those questions. I know some of them may have been difficult. Remember that no one in your community will ever know what you answered. Before we finish, I have a few more questions for you about how you felt during the survey.**		
O1	Do you feel that the questions you answered very easy to understand, easy to understand, difficult to understand, or very difficult to understand?	1=Very easy to understand<br>2=Easy to understand<br>3=Difficult to understand<br>4=Very difficult to understand<br>888=Don't Know<br>999=No Response	
O2	Overall, how honest would you say you were in answering the questions?	1=Not honest at all<br>2=Not very honest<br>3=Fairly honest<br>4=Very honest<br>5= Completely honest<br>888=Don't Know<br>999=No Response	
	This is the end of using the computer, so please call the interviewer over and return it to them		
**P. Post Interview Enumerator-only Questions**	**ONCE THE CHILD HAS FINISHED, TAKE THE ACASI TABLET BACK. CHECK THAT EVERYTHING LOOKS FINE AND THEN READ THE STATEMENT BELOW:**<br><br>**Thank you for answering the questions. You did a great job using the computer and answering questions about difficult issues.**<br><br>**PLEASE DEBRIEF WITH THE GIRL. THANK HER FOR HER TIME AND HONESTY IN ANSWERING THE QUESTIONS. TELL HER THAT YOU REALIZE THE QUESTIONS WERE DIFFICULT AND ANSWERING THEM WAS NOT EASY. ASSURE HER THAT HER RESPONSES ARE CONFIDENTIAL. INFORM HER THAT SHE CAN CONTACT YOU OR OTHER MEMBERS OF THE RESEARCH TEAM AT ANY TIME WITH QUESTIONS OR CONCERNS.**<br><br>**READ THE FOLLOWING: The place we are in right now belongs to the community-based organization (ADD NAME OF CBO HERE), in which there are women who provide psychosocial support. One of these women is in the area right now and I can take you directly to her if you would like. Otherwise, you can come back here at any time to speak with one of these women, or I can also give you her phone number.**<br><br>**LEAVE THE CARE FACILITIES CONTACT LIST WITH HER. IF SHE DOES NOT WANT TO TAKE THE LIST, INFORM HER THAT SHE CAN CONTACT THE RESEARCH TEAM AT ANY TIME FOR ASSISTANCE.**		

fGirl survey (ETH)			
**Question #**	**Question**	**Response options**	**Instructions**
	**ASK HER IF SHE HAS ANY FURTHER QUESTIONS.**<br><br>**ENUMERATOR, PLEASE COMPLETE THESE QUESTIONS BEFORE YOU FINALIZE THE SURVEY.**		
P4	Was anyone else present during the interview?	1=Yes<br>2=No	If Yes, answer P4b and P4c<br><br>If No, skip to P5
P4b	How much do you feel the presence of this person influenced the answers given?	1= A great deal<br>2= Some<br>3= Very little<br>4= None	
P5	Was the interview interrupted for any reason?	1=Yes<br>2=No	If Yes, answer P5b and P5c<br><br>If No, skip to P6
P5b	In your view, did it affect the interview?	1 = Yes<br>2 = No	
P6	What was the setting in which the interview took place?	1= Quiet, private<br>2= Some noise, semi-private<br>3= Noisy, people around	
P7	Does the respondent need a referral to any service providers?	1 = Yes<br>2 = No	
	**END OF SURVEY, GREAT WORK!**		
